# *CBL* mutations in chronic myelomonocytic leukemia often occur in the RING domain with multiple subclones per patient: Implications for targeting

**DOI:** 10.1371/journal.pone.0310641

**Published:** 2024-09-19

**Authors:** Kelly Lim, Winnie L. Kan, Pramod C. Nair, Monika Kutyna, Angel F. Lopez, Timothy Hercus, David M. Ross, Steven Lane, Chun Yew Fong, Anna Brown, Agnes Yong, David Yeung, Timothy Hughes, Devendra Hiwase, Daniel Thomas

**Affiliations:** 1 Discipline of Medicine, Adelaide Medical School, Faculty of Health and Medical Sciences, The University of Adelaide, Adelaide, SA, Australia; 2 Precision Cancer Medicine Theme, South Australian Health and Medical Research Institute (SAHMRI), University of Adelaide, Adelaide, SA, Australia; 3 Cytokine Receptor Laboratory, SA Pathology, Adelaide, SA, Australia; 4 College of Medicine and Public Health, Flinders Health and Medical Research Institute, Flinders University, Adelaide, SA, Australia; 5 SA Pathology, Adelaide, SA, Australia; 6 Department of Hematology and Bone Marrow Transplantation, Royal Adelaide Hospital, Adelaide, SA, Australia; 7 QIMR Berghofer Medical Research Institute, Brisbane, QLD, Australia; 8 Austin Health, Heidelberg, VIC, Australia; 9 Royal Perth Hospital, Perth, WA, Australia; 10 The University of Western Australia Medical School, Perth, WA, Australia; George Washington University, UNITED STATES OF AMERICA

## Abstract

Chronic myelomonocytic leukemia (CMML) is a rare blood cancer of older adults (3 in every 1,000,000 persons) characterized by poor survival and lacking effective mutation-specific therapy. Mutations in the ubiquitin ligase Cbl occur frequently in CMML and share biological and molecular features with a clonal disease occurring in children, juvenile myelomonocytic leukemia (JMML). Here we analyzed the clinical presentations, molecular features and immunophenotype of CMML patients with *CBL* mutations enrolled in a prospective Phase II clinical trial stratified according to molecular markers. Clinically, *CBL* mutations were associated with increased bone marrow blasts at diagnosis, leukocytosis and splenomegaly, similar to patients harboring *NRAS* or *KRAS* mutations. Interestingly, 64% of patients presented with more than one *CBL* variant implying a complex subclonal architecture, often with co-occurrence of *TET2* mutations. We found *CBL* mutations in CMML frequently clustered in the RING domain in contrast to JMML, where mutations frequently involve the linker helix region (*P*<0.0001). According to our comparative alignment of available X-ray structures, mutations in the linker helix region such as Y371E give rise to conformational differences that could be exploited by targeted therapy approaches. Furthermore, we noted an increased percentage of CMML CD34^+^ stem and progenitor cells expressing CD116 and CD131 in all *CBL* mutant cases and increased CD116 receptor density compared to healthy controls, similar to CMML overall. In summary, our data demonstrate that *CBL* mutations are associated with distinct molecular and clinical features in CMML and are potentially targetable with CD116-directed immunotherapy.

## Introduction

Monocytes give rise to tissue macrophages that can perform a myriad of biological functions ranging from innate immune activation to phagocytosis and wound healing. Chronic myelomonocytic leukemia (CMML) is a rare blood cancer of older adults (3 in every 1,000,000 persons) [1, 2] characterized by an increase in clonal CD14^+^ CD16^–^ classical monocytes and their precursors in the blood and bone marrow [3, 4]. Because of its subtle presentation, the disease is often diagnosed late, but is likely to rise in prevalence due to routine uptake of next-generation sequencing, lower threshold monocytosis criteria by the World Health Organization and increasing recognition by physicians, especially in persons previously treated with cytotoxic therapy (therapy-related CMML) [5, 6]. Many patients present with autoinflammatory features such as vasculitis, polychondritis, Sweet syndrome and pleural/pericardial effusions [7–10] but the mechanism and intersection of clonal monocytes and innate *vs*. adaptive immunity is not understood.

The molecular pathogenesis of CMML is only beginning to be characterized. Though 70% of CMML patients present without any cytogenetic abnormalities [11, 12] they harbor somatic mutations in genes that influence epigenetic regulation (*TET2*, *DNMT3A*, *ASXL1*, *EZH2*, *IDH1*, *IDH2*), mRNA splicing (*SRSF2*, *U2AF1*), genome stability (*SETBP1*, *TP53*), transcription regulation (*RUNX1*, *CEBPA*, *NPM1*) and cell signaling pathways (*KRAS*, *NRAS*, *CBL*, *PTPN11*, *JAK2*, *MPL*) [13–17]. Interestingly, CMML shares some biological and morphological features with a clonal disease occurring in young children, juvenile myelomonocytic leukemia (JMML). Around 90% of cases of JMML are associated with mutations in the RAS signaling pathway (*PTPN11*, *NRAS*, *KRAS*, *NF1* and *CBL*) [18–21]. Notably, while the overall molecular patterns of CMML and JMML are distinct, mutations in *CBL* are found with equal frequency, at approximately 15% [17, 18, 22, 23], in both diseases. Both CMML and JMML display hypersensitivity to the pro-inflammatory cytokine granulocyte-macrophage colony-stimulating factor (GM-CSF) which promote the differentiation of classical pro-inflammatory monocytes [24, 25]. Emerging reports suggest that *CBL* mutations are associated with inferior survival in both CMML and JMML [15, 26].

The *CBL* gene is located on 11q23.3 and encodes an E3 ubiquitin ligase (c-Cbl or Cbl) that acts as both a positive and negative regulator in the signal transduction of activated receptor tyrosine kinases (RTKs) and cytokine receptors. Cbl relays signals downstream of activated RTKs by functioning as an adaptor [27–30], and at the same time, attenuates signaling by promoting the ubiquitination of RTKs through its E3 ligase activity, marking them for degradation by the proteasome or via endocytosis [31–33]. Cbl recognizes phosphorylated tyrosines on active RTKs through its Src homology 2 domain within the tyrosine kinase binding domain (TKBD), and binds E2 ubiquitin-conjugating enzymes though its conserved RING domain. The TKBD and RING domain are connected by a 28 amino acid sequence, referred to as the linker helix region (LHR). A proline-rich region, serine-rich region, several C-terminal principal phosphorylation sites (Y674, Y700, Y731, Y774) and a ubiquitin-association domain (UBA) complete the structure of Cbl. Several studies on the role of the LHR and RING domain have shown that mutation of these domains, could lead to loss of activity and/or gain of oncogenicity [34–42].

To date, there has not been significant mutation-specific therapy developed for CMML, unlike chronic myeloid leukemia, and standard-of-care with hypomethylating agents azacitidine or decitabine is not curative [16, 43–47]. While allogeneic hematopoietic stem cell transplantation may be potentially curative, stem cell transplantation is not a viable option for most older CMML patients [48, 49]. Currently, survival is estimated at a median of 31 months, with even shorter life expectancy for patients with CMML-2 (more than 10% bone marrow blasts/promonocytes). Transformation to acute myeloid leukemia (AML), an aggressive cancer with poor long-term survival, occurs in up to 20% of CMML patients within 5 years [50–52].

In this study, we analyzed the clinical presentation of CMML patients with *CBL* mutations enrolled in a prospective clinical trial. Strikingly, 7 out of 11 (64%) patients were found to have more than one *CBL* clone, implying a complex clonal architecture. Clinically, *CBL* mutations were associated with a more proliferative phenotype evidenced by increased bone marrow blasts, leukocytosis and splenomegaly, similar to other RAS pathway mutations such as *KRAS*, *NRAS* and *PTPN11*. We also found that CMML *CBL* mutations often co-occurred with *TET2* mutations and were enriched in the RING domain compared to the LHR (*P*<0.0001). Furthermore, we noted an increased percentage of CD116 and CD131-expressing CMML CD34^+^ progenitors compared to healthy controls. In summary, our data suggest that *CBL* mutants are associated with distinct clinical and molecular features in CMML.

## Materials and methods

### Patients

Between 1 October 2021 and 30 September 2023, 24 patients with CMML, diagnosed according to the 2016 WHO Classification of Myeloid Neoplasms [53], who met the eligibility criteria (untreated CMML with high white cell count, cytopenia or constitutional symptoms) were enrolled in the trial with written, informed consent. Detection of *TET2*, *KRAS*, *NRAS* or *CBL* mutation at a variant allele frequency (VAF) percentage of ≥3% was a key inclusion criteria. Of these patients, 13 were male and 11 were female. The median age was 73 years (range 56−86 years). Written informed consent for genetic analysis and use of laboratory results and samples for scientific research were obtained during trial enrolment. The trial was approved in multiple centers across Australia including the Royal Adelaide Hospital, Royal Brisbane and Women’s Hospital and Austin Health. The trial was conducted with approval from the Central Adelaide Local Health Network Human Research Ethics Committee (2021/HRE00017) and registered on the Australian and New Zealand Clinical Trials Registry (ANZCTR) (Registration number ACTRN12621000223831, Acronym PREACH-M).

### Clinical presentation

All clinical and laboratory data were acquired at screening, prior to commencement of the therapy protocol outlined in the trial. Complete blood examination and bone marrow (BM) analyses were performed for each patient. The spleen craniocaudal length was determined by ultrasonography.

### Mutation screening

Targeted enrichment of selected coding exons and flanking intronic regions of 46 genes was performed using a custom-designed hematological neoplasms capture panel (Integrated DNA Technologies; HaemV1) and analyzed by next-generation sequencing (NGS) (Illumina NextSeq sequencing system). Variant calling was performed using Vardict and Mutect2 where variants with VAF <5% were reported where clinically significant. These assays were performed by accredited pathology laboratories across Australia.

### Cord blood and peripheral blood mononuclear cells from healthy donors

Umbilical cord blood was collected with written informed consent from scheduled cesarean section deliveries at the Women’s Health Unit, Lyell-McEwin Hospital (Adelaide, South Australia) between 12 November 2020 to 31 December 2023 with approval from the Women’s and Children’s Health Network Human Research Ethics Committee (HREC/20/WCHN/65; 2020/HRE01664). Peripheral blood buffy coat samples obtained with written informed consent was retrieved on 29 May 2023 and studies were approved by the Central Adelaide Local Health Network Human Research Ethics Committee (HREC/15/RAH/448) and conducted in accordance with the Declaration of Helsinki. Samples were processed by density gradient centrifugation using Lymphoprep^TM^ (Stemcell Technologies, USA) to isolate mononuclear cells (MNCs).

### Flow cytometry

Peripheral blood mononuclear cells (PB-MNCs) were collected at baseline from patients in the PREACH-M trial. 2–3 *CBL* mutant and 2–3 *CBL* wildtype CMML samples (*n* = 4–6) were immunophenotyped by spectral flow cytometry (Cytek Aurora, USA). Control samples include cord blood mononuclear cells (CB-MNCs) (*n* = 2) and PB-MNCs from healthy donors (*n* = 1). All MNCs were incubated with Human TruStain FcX^TM^ (BioLegend, USA) and True-Stain Monocyte Blocker^TM^ (BioLegend, USA) prior to antibody staining. Antibody panel included mouse anti-human CD45 (HI30) BV421, CD14 (M5E2) PE-Cy7, CD16 (3G8) PE-Cy5, CD34 (8G12) APC, CD114 (LMM741) PE, CD116 (hGMCSFR-M1) PE, CD131 (1C1) PE; CD131 (3D7) BV421 and rat anti-human CD115 (9-4D2-1E4) PE. Viability was determined using Zombie Aqua^TM^ Fixable Dye (BioLegend, USA). A list of catalogue numbers and clones of antibodies can be found in **S1 Table**.

### Hotspot and protein structure analysis

Data from the Catalogue of Somatic Mutations in Cancer (COSMIC) were analyzed (last accessed on 15 February 2024). Analysis of *CBL* mutations were filtered as follows: Primary site hematopoietic and lymphoid, histology hematopoietic neoplasm; sub-histology CMML or JMML. 120 variants were found for CMML, 46 for JMML. Variants were stratified to include nonsense or missense substitutions, frameshift insertion or deletions, and in-frame insertions or deletions, with the exclusion of synonymous mutations, within the coding sequence. 17 coding region variants were detected in the PREACH-M cohort, making the total variants for CMML 137. The six most common mutations for CMML and JMML were identified. Protein structures were sourced from the Protein Data Bank (PDB) and analyzed using Maestro 13.8 (Schrodinger, USA) and UCSF ChimeraX (UCSF Resource for Biocomputing, Visualization and Information, USA). RMSD calculations were performed without rejection of any atoms during fit and distance was measured between alpha carbons of residue 227 and 371 of Cbl.

### Statistical analysis

Values between unique samples were presented as mean ± standard error of margin (S.E.M.) or as mean ± standard deviation (S.D.) between technical replicates. For comparisons between groups, Student’s *t*-test or Mann-Whitney test was applied to analyze measurement (continuous) data and Fisher’s exact test for enumeration (categorical) data. All statistical analyses were performed using GraphPad Prism 10. *P*-values for Student’s *t*-tests were two-tailed, Mann-Whitney tests were one-tailed, and Fisher’s exact tests were two-tailed. *P*<0.05 was considered statistically significant.

## Results

### *CBL* mutations are associated with increased marrow blasts, leukocytosis and splenomegaly, consistent with RAS pathway activation

**Fig 1:**
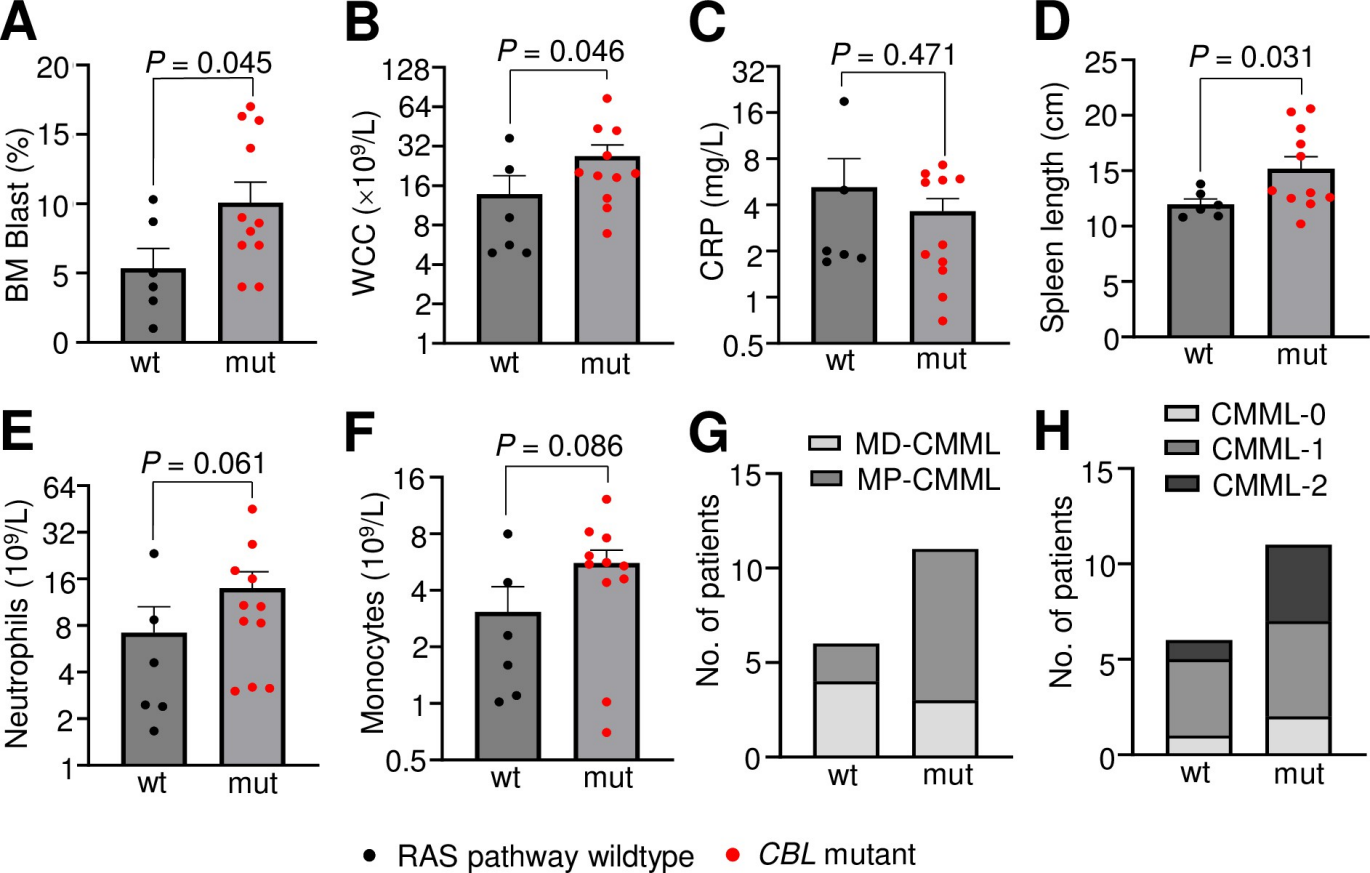
*CBL* mutants are associated with proliferative features, increased BM blast percentage, leukocytosis and splenomegaly. (**A-F**) Clinical characteristics of PREACH-M cohort at baseline, stratified based on the detection of *CBL* mutation. (**G**) MD-, MP-CMML classification based on WCC^**α**^ (**H**) CMML-0, -1, -2 classifications based on BM blast percentage^**β**^. 2016 WHO Classification: ^**α**^Based on WCC: MD-CMML WCC<13×10^9^/L, MP-CMML WCC>13×10^9^/L. ^**β**^ Based on BM blast %: CMML-0 PB <2%, BM <5%; CMML-1 PB 2–4%, BM 5–9%, CMML-2 PB>5%, BM 10–19%. Bars represent mean ± standard error of mean. Mann-Whitney test used to determine statistical significance, where *P*<0.05 was statistically significant. [BM bone marrow; WCC white cell count; CRP C-reactive protein; MD-CMML myelodysplastic-CMML; MP-CMML myeloproliferative-CMML, wt wildtype, mut mutant].

Targeted NGS was performed on 24 *de novo* CMML baseline patient samples from the PREACH-M trial. Overall, RAS pathway (*KRAS*, *NRAS*, *PTPN11*, *CBL*) mutations were detected in 18/24 (75%) patients with *CBL* mutations detected in 11/24 (46%) patients (**Tables 1, 2** and **S2**). Consistent with previous findings where RAS pathway mutations were linked to the proliferative variant of CMML [54, 55], patients with RAS pathway mutations had increased BM blast percentage (9.2 ± 1.1 *vs*. 5.3. ± 1.4%, *P* = 0.05), white cell count (WCC) (30.4 ± 6.0 *vs*. 13.8 ± 5.3×10^9^/L, *P* = 0.02), neutrophil absolute count (15.2 ± 3.3 *vs*. 7.2 ± 3.4×10^9^/L, *P* = 0.03), monocyte absolute count (7.8 ± 1.7 *vs*. 3.1 ± 1.1×10^9^/L, *P* = 0.03) and spleen length (15.2 ± 0.9 *vs*. 12.0 ± 0.5 cm, *P* = 0.04) compared to patients wildtype for any RAS pathway mutations (**Table 1**). This finding led us to further stratify our cohort and indeed, patients with *CBL* mutations were also noted to have increased BM blast percentage (10.1 ± 1.5 *vs*. 5.3 ± 1.4%, *P* = 0.05), WCC (26.8 ± 5.9 *vs*. 13.8 ± 5.2×10^9^/L, *P* = 0.05), and spleen length (15.2 ± 1.1 *vs*. 12.0 ± 0.5 cm, *P* = 0.03) compared to *CBL* wildtype patients without RAS pathway mutations (**Table 2** and **Fig 1A–1F**). Comparisons between *CBL* mutant/RAS pathway wildtype *vs*. RAS pathway mutant/*CBL* wildtype cases revealed consistent trends (**S1 Fig**). 10/11 (90.9%; *P* = 0.03) patients with *CBL* variants presented with splenomegaly (**S3 Table**). This underscores the strong proliferative phenotype conferred by mutations in *CBL* in CMML. Importantly, 8/11 (73%) *CBL* variants were in cases classified as myeloproliferative-CMML (MP-CMML) based on WCC (**Fig 1G**), and 9/11 (82%) classified as CMML-1 or -2 based on BM blast percentage (**Fig 1H**) according to the 2016 WHO classification, linking *CBL* mutations not just to a proliferative phenotype but to more advanced stages of the disease, and therefore, to increased risk of progression to AML [50].

**Table 1:** Clinical characteristics, complete blood examination and bone marrow analyses of CMML patients in the PREACH-M trial stratified as RAS pathway (*KRAS*, *NRAS*, *PTPN11*, *CBL*) mutant *vs*. wildtype.

Variable	Total (*n* = 24)	RAS pathway wildtype (*n* = 6)	RAS pathway mutant (*n* = 18)	*P-*value
Gender				
Male, *n* (%)	13 (54%)	2 (15%)	11 (85%)	0.3572
Female, *n* (%)	11 (46%)	4 (36%)	7 (64%)	
Age (years)				
Mean (range)	72 (56–86)	73 (56−86)	70 (56−79)	0.1424
WHO classification				
CMML-0, *n* (%)	4 (17%)	1 (17%)	3 (17%)	0.8215
CMML-1, *n* (%)	13 (54%)	4 (67%)	9 (50%)	
CMML-2, *n* (%)	7 (29%)	1 (17%)	6 (33%)	
MD-CMML, *n* (%)	9 (38%)	4 (67%)	5 (28%)	0.1501
MP-CMML, *n* (%)	15 (63%)	2 (33%)	13 (72%)	
BM Blast (%)				
Mean (range)	7.3 (1.0−17.0)	5.3 (1.0−10.3)	9.2 (2.0−17.0)	0.0493
WCC (×10^9^/L)				
Mean (range)	22.1 (4.9−103.3)	13.8 (4.9−36.8)	30.4 (6.9−103.3)	0.0224
Hb (g/L)				
Mean (range)	107 (79−143)	104 (79−124)	109 (82−143)	0.3426
PLT (×10^9^/L)				
Mean (range)	82 (7−219)	91 (7−219)	73 (18−192)	0.3235
Neutrophils (10^9^/L)				
Mean (range)	11.2 (1.67−47.5)	7.2 (1.7−23.3)	15.2 (3.0–47.5)	0.0329
Monocytes (10^9^/L)				
Mean (range)	5.4 (0.7−32.8)	3.1 (1.0−8.0)	7.8 (0.7−32.8)	0.0267
CRP (mg/L)				
Mean (range)	4.9 (0.6−18.9)	5.2 (1.7−18.9)	4.5 (0.6−17)	0.4796
Spleen craniocaudal length (cm)				
Mean (range)	13.6 (9.8−20.6)	12.0 (10.8−13.8)	15.2 (9.8−20.6)	0.0400

*n* number of patients; BM bone marrow; WCC white blood cell count; Hb haemoglobin; PLT platelet; CRP C-reactive protein; MD-CMML myelodysplastic CMML; MP-CMML myeloproliferative CMML.

Mann-Whitney test was applied to continuous and Fisher’s exact test to categorical data for statistical analysis where *P*<0.05 was statistically significant.

2016 WHO Classification:

(i) Based on BM blast %: CMML-0 PB <2%, BM <5%; CMML-1 PB 2–4%, BM 5–9%, CMML-2 PB>5%, BM 10–19%.

(ii) Based on WCC: MD-CMML WCC<13×10^9^/L, MP-CMML WCC>13×10^9^/L

**Table 2:** Clinical characteristics, complete blood examination and bone marrow analyses of CMML patients in the PREACH-M trial stratified as *CBL* mutant *vs*. RAS pathway wildtype.

Variable	Total (*n* = 17)	RAS pathway wildtype (*n* = 6)	*CBL* mutant (*n* = 11)	*P*-value
Gender				
Male, *n* (%)	8 (47%)	2 (33%)	6 (55%)	0.6199
Female, *n* (%)	11 (65%)	4 (67%)	5 (45%)	
Age (years)				
Mean (range)	72 (56−86)	73 (56−86)	71 (56−79)	0.2539
WHO classification				
CMML-0, *n* (%)	3 (18%)	1 (17%)	2 (18%)	0.7964
CMML-1, *n* (%)	9 (53%)	4 (67%)	5 (45%)	
CMML-2, *n* (%)	5 (29%)	1 (17%)	4 (36%)	
MD-CMML, *n* (%)	7 (41%)	4 (67%)	3 (27%)	0.1618
MP-CMML, *n* (%)	10 (59%)	2 (33%)	8 (73%)	
BM Blast (%)				
Mean (range)	7.7 (1.0−17.0)	5.3 (1.0−10.3)	10.1 (4.0−17.0)	0.0457
WCC (×10^9^/L)				
Mean (range)	20.3 (4.9−74.1)	13.8 (4.9−36.8)	26.8 (6.9−74.1)	0.0462
Hb (g/L)				
Mean (range)	106 (79−128)	104 (79−124)	107 (91−128)	0.4893
PLT (×10^9^/L)				
Mean (range)	89 (7−219)	91 (7−219)	88 (27−192)	0.5000
Neutrophils (10^9^/L)				
Mean (range)	10.6 (1.7−45.2)	7.2 (1.7−23.3)	14.0 (3.0−45.2)	0.0608
Monocytes (10^9^/L)				
Mean (range)	4.3 (0.7−12.2)	3.1 (1.0−8.0)	5.6 (0.7−12.2)	0.0859
CRP (mg/L)				
Mean (range)	4.4 (1.7−18.9)	5.2 (1.7−18.9)	3.6 (0.7−7.3)	0.4708
Spleen craniocaudal length (cm)				
Mean (range)	13.6 (10.2−20.6)	12.0 (10.8−13.8)	15.2 (10.2−20.6)	0.0308

*n* number of patients; BM bone marrow; WCC white blood cell count; Hb haemoglobin; PLT platelet; CRP C-reactive protein; MD-CMML myelodysplastic CMML; MP-CMML myeloproliferative CMML

Mann-Whitney test was applied to continuous and Fisher’s exact test to categorical data for statistical analysis where *P*<0.05 was statistically significant.

2016 WHO Classification:

Based on BM blast %: CMML-0 PB <2%, BM <5%; CMML-1 PB 2–4%, BM 5–9%, CMML-2 PB>5%, BM 10–19%.

Based on WCC: MD-CMML WCC<13×10^9^/L, MP-CMML WCC>13×10^9^/L

### *CBL* mutations co-occur frequently with *TET2*

**Fig 2:**
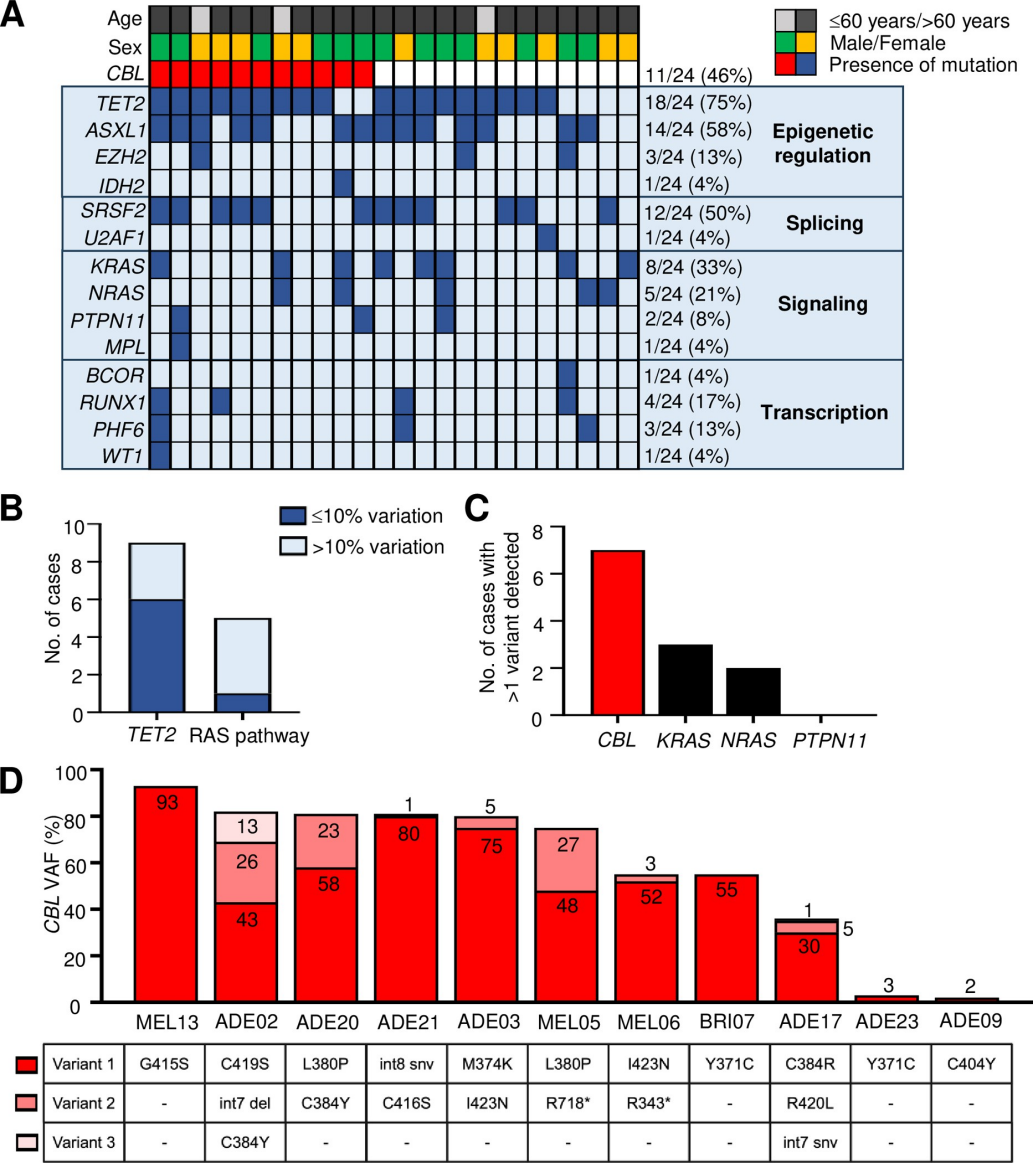
*CBL* mutants frequently co-occur with *TET2* mutants and are associated with a complex subclonal architecture. (**A**) Oncoplot for the PREACH-M cohort (*n* = 24). Mutation groups are shown in rows with each individual patient represented by a column. The presence of a mutation is indicated by the red or blue colored bars. Age category of the patients indicated by the black and grey bars and sex of patients by the green and gold bars. (**B**) Number of *CBL* mutant cases where *TET2* mutations (*n* = 9) and other RAS pathway mutations (*n* = 5) were detected, where variation in the VAF of *CBL vs*. *TET2* or RAS pathway mutant clones were ≤10% (dark blue) or >10% (light blue) (**C**) Number of cases where more than one variant of *CBL*, *NRAS*, *KRAS* or *PTPN11* mutation was detected. (**D**) Details of *CBL* variants detected in each patient with *CBL* mutation. [VAF variant allele frequency].

Of the 24 patients in the study, 75% (18/24) were detected to have *TET2* mutation, 58% (14/24) *ASXL1*, 50% (12/24) *SRSF2* and 46% (11/24) *CBL* (**Fig 2A**). Of the 11 patients with *CBL* mutation, 9 (82%) had co-occurring *TET2* mutation. In 67% (6/9) of instances when these mutations are detected in the same patient, the difference in VAF magnitude between *CBL* and *TET2* were ≤10% (MEL13, ADE02, ADE20, MEL05, MEL06, BRI07) (**Figs 2B and S2**), indicating these mutations may co-occur within the same clone. On the contrary, mutations in *CBL* and other RAS pathway genes (*KRAS*, *NRAS*, *PTPN11*) are not only infrequently found in the same patient (5/11; 45%), the VAF of the dominant *CBL* or RAS pathway mutant clone tend to be discordant, with VAF differences >10% (MEL13, BRI07, ADE17, ADE09), except in one case where *CBL* and *PTPN11* VAF were ≤3% (**Figs 2B and S2**).

### Multiple *CBL* mutant subclones found in CMML

Strikingly, in 7/11 (64%) patients with *CBL* mutation, more than one *CBL* variant can be detected (**Fig 2C**). Of these, 2 patients had 3 variants while 5 patients had 2 variants (**Fig 2D**). In comparison, multiple subclones within a patient are more uncommon in *KRAS* (3/8; 38%), *NRAS* (2/5; 40%) and *PTPN11* (0/3; 0%) mutant cases (**Fig 2C**).

### CMML linked to high CD116 and CD131 in the progenitor subpopulation

**Fig 3:**
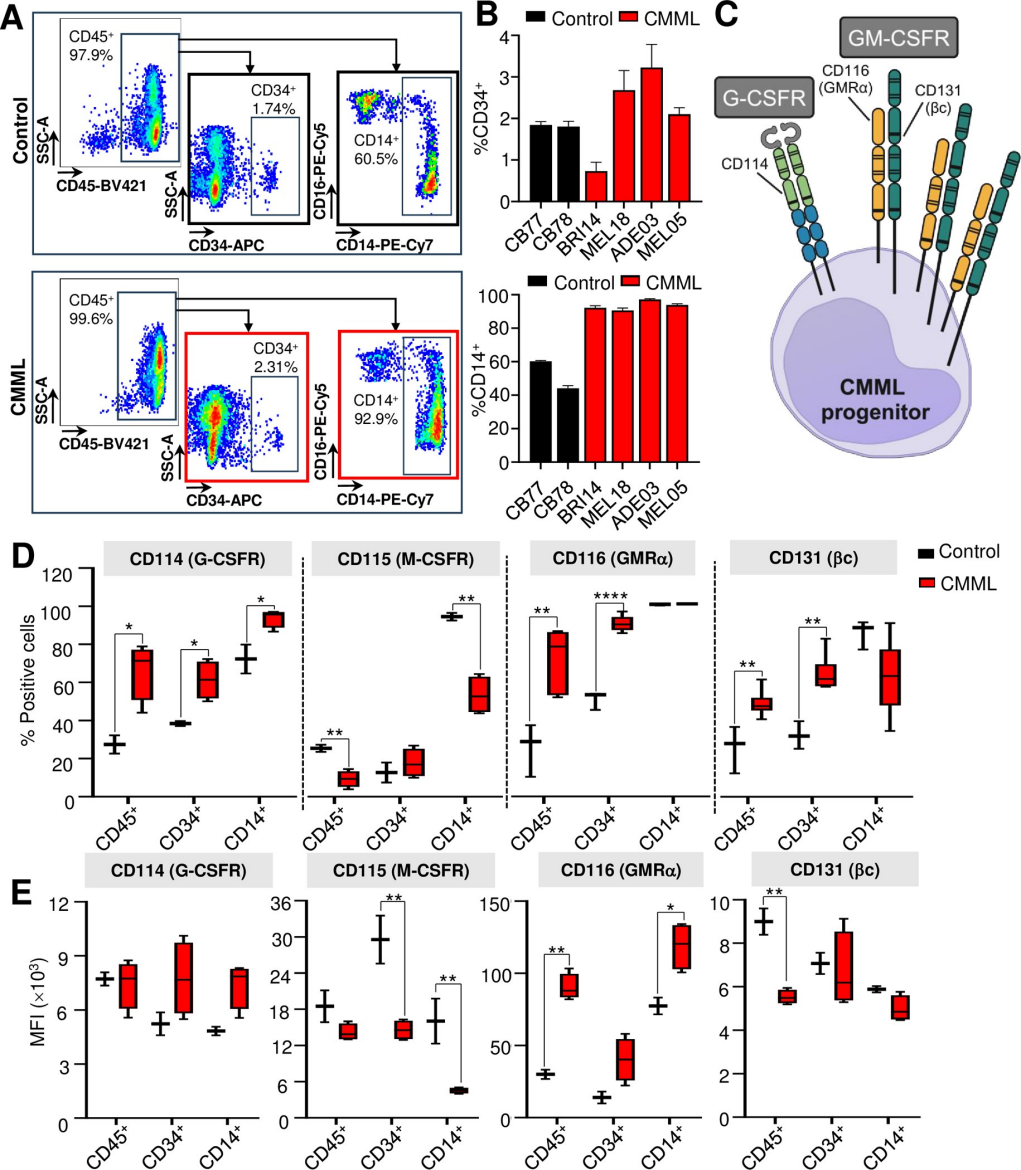
CMML have an increased percentage of CD116 and CD131 positive CD34^+^ stem and progenitor cells. (**A**) Flow cytometry analysis of a representative CMML sample and healthy control stained for CD45, CD34, CD14 and CD16, and gating strategy used to define CD45^+^ mononuclear cells, CD34^+^ stem and progenitor cells and CD14^+^ monocytes. (**B**) Percentage of CD34^+^ progenitors and CD14^+^ monocytes in CMML samples (*n* = 4) *vs*. healthy control (*n* = 2). (**C**) Illustration of the cluster of differentiation (CD) markers where CD114 is a marker for G-CSFR, CD116 GMRα and CD131 βc. G-CSFR is homodimeric while GM-CSFR is heterodimeric receptor consisting of GMRα and βc. The expression of CD114, CD115, CD116 and CD131 in CMML samples (*n* = 4–6) *vs*. control (*n* = 2–3) in CD45^+^, CD34^+^ and CD14^+^ subpopulations, expressed as percentage of positively stained cells (**D**) and MFI (**E**) compared to control (cord blood or peripheral blood mononuclear cells from healthy donors). Bars represent mean ± standard deviation in (**B**). Box and whiskers graphs were plotted with min and max in (**C**) and (**D**). Unpaired Student’s *t*-test between CMML *vs*. healthy control used to determine statistical significance, where *P*<0.05 was statistically significant. *P<0.05, **P<0.01, ***P<0.001, ****P<0.0001. [MFI mean fluorescence intensity; G-CSFR granulocyte-colony stimulating factor receptor; GMRα granulocyte-macrophage colony stimulating factor receptor subunit α; βc beta common subunit].

As CMML is a disease characterized by upregulation of inflammatory cytokines [56, 57] and expansion of pro-inflammatory granulocyte-macrophage-like progenitor cells and monocytes with enhanced cytokine receptor signaling [58, 59], we analyzed the immunophenotype of primary patient samples (*n* = 2–3 *CBL* mutant; *n* = 2–3 *CBL* wildtype) focusing on cytokine receptor expression, including granulocyte colony-stimulating factor receptor (G-CSFR, CD114), macrophage colony-stimulating factor receptor (M-CSFR, CD115) and the heterodimeric granulocyte-macrophage colony-stimulating factor receptor (GM-CSFR) comprising the alpha subunit (GMRα, CD116) and the beta common subunit (βc, CD131), in the CD45^+^ MNCs, CD34^+^ hematopoietic stem and progenitor cells and CD14^+^ monocytes (gating strategy outlined in **Fig 3A**). CMML patient samples had higher percentage of CD14^+^ cells compared to normal donors, consistent with expansion of monocytes and clinical presentation of the disease (**Fig 3B**).

Our data revealed that the percentage of cells in CMML patient samples expressing CD116 (GMRα) was significantly higher in the total MNC population compared to control, and this was most pronounced in the CD34^+^ progenitor subpopulation (89.7 ± 1.6 *vs*. 50.3 ± 2.7%; *P* = 0.000003) (**Fig 3C** and **3D**). In contrast, the percentage of CD116 expressing cells was similar between healthy and CMML-derived CD14^+^ monocytes (**Fig 3D**). Interestingly, the density of CD116 expression represented by mean fluorescence intensity (MFI) was upregulated in our CMML cohort *vs*. healthy controls (CD45^+^ 90.3 ± 4.6×10^3^ *vs*. 30.0 ± 3.2×10^3^, *P* = 0.001; CD34^+^ 40.2 ± 7.4×10^3^ *vs*. 13.9 ± 4.1×10^3^, *P* = 0.08; CD14^+^ 118.9×10^3^ ± 8.1×10^3^ *vs*. 77.4×10^3^ ± 5.8×10^3^; *P* = 0.03) (**Fig 3E**). We also noted an increase in the percentage of CD131 expression in the MNCs, particularly in the CD34^+^ progenitors in CMML compared to control (64.3 ± 3.8 *vs*. 32.1 ± 4.1%; *P* = 0.001), although not in terms of MFI (**Fig 3D** and **3E**). We did not observe a difference in receptor expressions between *CBL* mutant and *CBL* wildtype CMML patient samples (**S3 Fig**).

We also noted increases in the percentage of CD114^+^ cells across all CMML cell populations (CD45^+^ *P* = 0.03; CD34^+^ *P* = 0.04; CD14^+^ *P* = 0.02), with no difference in the MFI (**Fig 3C** and **3D**). In contrast, the percentage of CD115^+^ cells was notably lower in the CMML CD45^+^ MNCs (*P* = 0.01) and CD14^+^ monocytes (*P* = 0.005), with reductions in MFI seen in both CD34^+^ (*P* = 0.005) and CD14^+^ (*P* = 0.007) populations (**Fig 3D** and **3E**).

### *CBL* mutations are enriched in the RING domain in CMML compared to JMML

**Fig 4:**
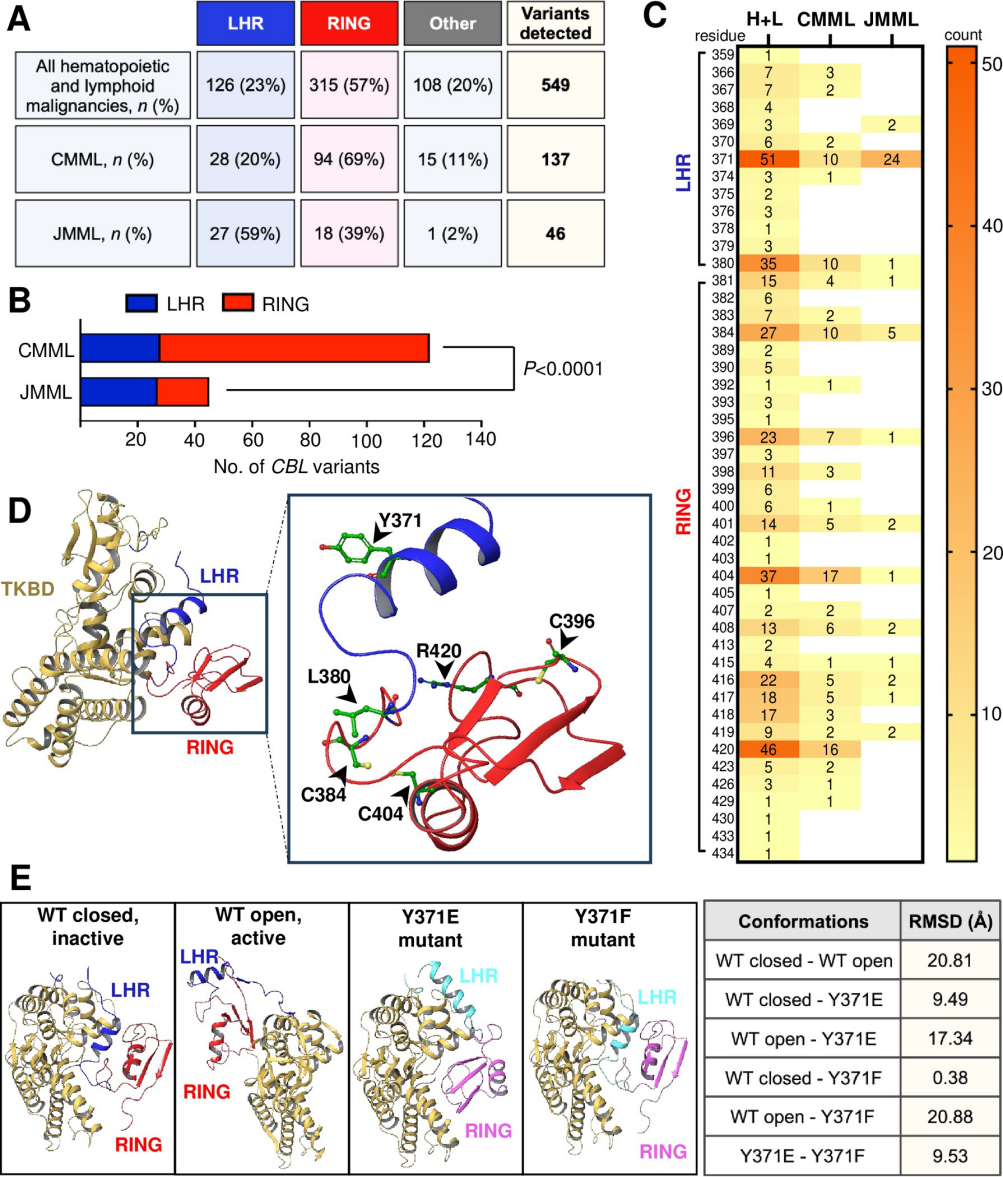
*CBL* mutation hotspots in CMML cluster in the RING domain, unlike in JMML where they more commonly occur within the LHR. (**A**) Table of *CBL* variants detected in our PREACH-M cohort combined with data sourced from COSMIC. Variants include nonsense or missense substitutions, frameshift and in-frame insertions or deletions within the coding sequence of *CBL*, filtered for all hematopoietic and lymphoid malignancies including CMML and JMML (*n* = 549), and CMML only (*n* = 137) or JMML only (*n* = 46) (**B**) Contingency analysis of *CBL* mutation hotspots within the LHR and RING domain of Cbl in CMML and JMML (**C**) Heat map representation of all sites within the LHR (amino acid residues 353–380) and RING domain (amino acid residues 381–435) where mutations have been reported. Numbers within the figure and on the scale depict counts (**D**) Tertiary protein structure of native wildtype Cbl (PDB ID 2Y1M) in inactive, closed conformation. The TKBD is colored beige, LHR blue and RING domain red. Amino acid residues of the top 6 mutation hotspots are indicated in inset; Tyrosine 371 (Y371), Leucine 380 (L380), Cysteine 384 (C384), Cysteine 396 (C396), Cysteine 404 (C404) and Arginine 420 (R420). (**E**) X-ray structures of wildtype Cbl in unphosphorylated, inactive state and in closed conformation (PDB ID 2Y1M), wildtype Cbl in Y371 phosphorylated, active state and in open conformation (PDB ID 4A4C), mutant Cbl Y371E (PDB ID 5HKX) and mutant Cbl Y371F (PDB ID 5J3X). The TKBD is colored beige, LHR of wildtype blue, LHR of mutant cyan, RING domain of wildtype red, RING domain of mutant pink. RMSD values between various Cbl conformations are shown in table. Statistical analysis was performed using two-sided Fisher’s exact test, where *P*<0.05 was statistically significant. [TKBD tyrosine kinase binding domain; LHR linker helix region; RING RING domain; H+L all hematopoietic and lymphoid malignancies; WT wildtype; RMSD root mean square deviation (distanced-based measure of protein structure similarity)].

We then combined the new mutation data from our cohort with the publicly available data in COSMIC to assess which domains of Cbl were commonly perturbed. We found that *CBL* mutations in CMML and JMML are concentrated within the coding sequence of the LHR and RING domain of Cbl (**Fig 4A**). Furthermore, we noted that in CMML, mutations most frequently occur within the RING domain (amino acid residues 381–435) contrary to JMML, where mutations within the LHR (amino acid residues 353–380) are most common (*P*<0.0001) (**Fig 4B**).

The 6 most common *CBL* mutations in all hematopoietic and lymphoid malignancies occur in codons affecting amino acid residues 371, 380, 384, 396, 404, 420 (**Fig 4C** and **4D**). In the PREACH-M cohort in particular, mutations in residue 384 were detected 3 patients, 371 in 2 patients, 380 in 2 patients, 404 and 420 in 1 patient, respectively. In CMML, missense substitutions cysteine 404 to tyrosine (C404Y) (13/137; 10%) and arginine 420 to glutamine (R420Q) (12/137; 9%) were most common, while in JMML, tyrosine 371 to histidine (Y371H) substitution was most common (21/46; 46%).

### Mutations at residue 371 within the LHR can result in novel conformational change

Finally, we performed comparative structural alignments of available mutant Cbl structures resolved by X-ray diffraction publicly available via the Protein Data Bank (PDB). Comparison of the LHR of wildtype Cbl protein in the closed, inactive conformation (Y371 unphosphorylated) (PDB 2Y1M) [40] against the open, active conformation (Y371 phosphorylated) (PDB 4A4C) [40] (**Fig 4E**), revealed a root mean square deviation (RMSD) of 20.81Å, indicating that a significant conformational change takes place when Cbl becomes activated by phosphorylation. Further, we also compared PDB structures 2Y1M and 4A4C with structures comprising tyrosine 371 to glutamic acid (Y371E) (PDB 5HKX) and tyrosine 371 to phenylalanine (Y371F) (PDB 5J3X) [41] LHR mutations. Interestingly, we inferred that the Y371E mutant Cbl possesses an entirely different conformation to either closed, inactive or open, active wildtype Cbl (RMSD 9.49Å and 17.34Å, respectively). We noted that replacement of the polar, bulky tyrosine with the negatively charged glutamic acid (Y371E) resulted in perturbation of the LHR-TKBD interface and subsequent total displacement of the LHR and RING domain compared to both inactive and active wildtype Cbl (**Fig 4E**). In contrast, when tyrosine was replaced with a structurally similar residue phenylalanine (Y371F), the LHR-TKBD interface was unperturbed, and the mutant closely mimicked the native, inactive state of wildtype Cbl (RMSD 0.38Å) and not the active state (RMSD 20.88Å) (**Fig 4E**). Additional measurements relating to the structural differences between wildtype and mutant Cbl can be found in **S4 Table**.

## Discussion

Our data, obtained from patients enrolled in a prospective multicenter interventional study, highlight several clinical and molecular features of *CBL* mutants in CMML. Our results are generally consistent with previous studies that performed next generation sequencing in CMML patients but we note a higher frequency of *CBL* variants (11 of 24 patients, 46%) than others report (12.8%) [60, 61], possibly due to strict trial eligibility criteria (higher white cell count or cytopenia). *CBL* variants were associated with a myeloproliferative phenotype, including higher white cell count and splenomegaly with many patients having increased blasts at diagnosis, similar to patients with other RAS pathway mutations. Notably, many patients harbored multiple *CBL* subclones (intrapatient molecular heterogeneity) that was not observed to the same extent for other RAS pathway mutations. This may be significant because subclonal abundance, especially a branched pattern of clonal evolution, is associated with a favorable outcome in AML [62].

We observed a strong overlap and clonal correlation between *CBL* mutations and *TET2* mutation. We noted another study that has found a modest association of *TET2* with *CBL* mutations (r<0.25; P<0.1) [60], despite the high frequency of *TET2* mutation overall. A number of *in vivo* murine studies have highlighted a role for TET2 in suppressing innate immune signaling in monocytes, with *TET2* mutant monocytes showing enhanced pro-inflammatory responses to stimuli such as lipopolysaccharide [63, 64]. It is plausible that the *CBL* mutation serves to further amplify innate immune signaling by preventing ubiquitination and turnover of cytokine and Toll-like receptors but the exact cellular compartments within which this occurs is not defined. Thus, it is significant that a high percentage of *CBL* mutant CMML CD34^+^ progenitors express GMRα (CD116, 89.7%) and its partner subunit, βc (CD131, 64.3%), suggesting that a substantial proportion of CD34^+^ cells are primed to respond to the cytokine GM-CSF (**Fig 3C–3E**). Indeed, we know from previous studies that CMML display hypersensitivity to GM-CSF akin to JMML, especially in cases that have RAS pathway mutations [24, 25]. In contrast, the percentage expression of the receptor for M-CSF (CD115) was decreased in CMML progenitors compared to controls, indicating this alternate monocyte cytokine is unlikely to be driving the disease. We also noted increased percentage of G-CSFR (CD114)-expressing CMML compared to controls, although not to the same extent as CD116, indicating CMML progenitors may also respond to G-CSF.

Previous studies with GM-CSF neutralizing antibody [25] and our own work with the GM-CSF E21R antagonist and successful engraftment of CMML patient samples in mice transgenic for human GM-CSF [24] provide strong evidence that GM-CSF is an essential growth factor for CMML *in vitro* and *in vivo*. Our findings that CD116 is upregulated in the CD34^+^ progenitors is interesting as it raises the question of the effect of GM-CSF on the leukemia-initiating cell population. This is in agreement with a recent study using single cell RNA-seq to map the differentiation trajectories of CD34^+^ progenitors in CMML primary patient samples [59] where the authors also showed the upregulation of CD116 in a cell cluster enriched for granulocyte/monocyte progenitor-like inflammatory hematopoietic stem and progenitor cells that may have self-renewal capacity in CMML patients with a monocyte-biased differentiation trajectory. Recently, we found that interleukin-3 (IL-3) receptor stoichiometry is a critical determinant in cell fate and IL-3 receptor overexpression in leukemia stem cells leads to biased activation of distinct transcriptional programs and signaling pathways to drive stemness programs *vs*. cell differentiation [65]. This propels us to hypothesize that although GM-CSF has been mostly associated with the proliferation and differentiation of hematopoietic progenitors and mature cells, it is possible that aside from the cytokine hypersensitivity previously shown [25], GM-CSF may also have unique signaling and effects on stem cell maintenance and function in CD116-overexpressing CD34^+^ progenitors for disease initiation and generation of the pro-inflammatory phenotype associated with this disease. Future studies should examine the kinetics of receptor turnover and phosphorylation peak and attenuation in this primary population, the signaling pathways involved and determine whether CD116 can be used to distinguish between *CBL* mutant leukemia *vs*. healthy stem cells. Indeed, the effects of anti-CD116 or anti-GM-CSF therapies (or in combination), on these populations warrant further investigation.

Cbl adopts a closed and open conformation dependent on Y371 phosphorylation to allow for the binding of ubiquitin-conjugating enzyme E2 [40]. Thus, it follows that the loss of this key tyrosine residue in position 371 within the LHR could have dramatic implications for the conformation and activity of Cbl. Tyrosine 371 is in a buried environment, where it forms a hydrogen bond with threonine 227 (T227) and makes several van der Waals interactions with residues in the hydrophobic pocket of the TKBD [36, 40], playing a structural role in maintaining the integrity of the LHR-TKBD interface, and importantly, in keeping Cbl in a closed conformation, autoinhibited state [40]. When Y371 is phosphorylated, an open conformation is adopted, and autoinhibition is abolished leading to Cbl becoming a more active ligase [35, 38, 40, 66, 67]. Our analysis of the X-ray structures demonstrates that Cbl conformation is sensitive to the amino acid residue at position 371. Indeed, our inference following comparative structural alignments is that, depending on the nature of the substituting residue, mutation at position 371 (such as Y371E, PDB 5HKX) not only results in the impairment of phosphorylation-dependent activation, but can also yield an entirely novel conformation of Cbl, different from both the inactive (unphosphorylated, closed) and active (phosphorylated, open) conformations. With this conformational drift, the RING domain and E2 enzyme cannot be in sufficient proximity to the substrate binding site of the TKBD for effective ubiquitination. Thus, ubiquitination and degradation of activated RTKs would be predicted to occur less efficiently, resulting in sustained downstream signaling that may contribute to oncogenicity and disease progression. In contrast, mutations that do not perturb the LHR-TKBD interaction (such as Y371F, PDB 5J3X [41]) would mimic the conformation of native, wildtype Cbl, albeit no longer capable of increased catalytic efficiency due to loss of the phosphorylation site. This is consistent with early evidence that Cbl Y371 mutants can exist in different states of activity depending on the chemical nature of the amino acid substitution [41].

In CMML, *CBL* mutations were found in both the LHR and RING domains of the protein but with significant enrichment for mutations in the RING domain compared to JMML. The RING domain determines the specificity of Cbl E3 for its cognate E2 enzyme, recognizes lysines to be ubiquitinated and serves as a scaffold for optimal orientation for ubiquitin transfer between E2 and its substrate RTK [36] but the structure of Cbl RING domain mutations has not been determined. Elucidation of distinct mutant Cbl conformations is significant because new drug development strategies could employ proteolysis targeting chimera (PROTAC) technology for targeted protein degradation of mutant Cbl with conformations different from wildtype. Conversely, a new Cbl-b inhibitor C7683 currently in phase I clinical trials for advanced solid tumor malignancies, designed to keep wildtype Cbl-b locked in an inactive state [68], may partially mimic LHR Cbl mutations and thus should be used with caution in patients with clonal hematopoiesis, CMML or JMML.

A limitation of our study is the relatively small number of *CBL* mutant positive cases, reflecting the rarity of CMML. Nevertheless, certain clinical and molecular features are consistent across the cohort and are congruent with data available in COSMIC, implying that *CBL* mutant CMML may have a characteristic phenotype. Studies with larger cohorts are required to distinguish *CBL* mutation CMML from other RAS pathway mutations such as *NRAS*, *KRAS* and *PTPN11*. To date, one study using serial VAF measurements did not show significant change in clone size for CMML patients, including *CBL* clones, treated with azacitidine alone suggesting epigenetic effects, rather than mutation-specific effects, were linked to therapeutic benefit [16]. In a phase I study, quizartinib inhibition of the receptor tyrosine kinase FLT3, a Cbl target for ubiquitination and internalization [69], did not appear to impact CMML with *CBL* mutations, suggesting phosphorylated FLT3 is not a critical substrate of Cbl in CMML [70]. Future work should examine the effect of CD116-targeted immunotherapy on the clonal dynamics of *CBL* RING domain *vs*. LHR mutants.

## Supporting information

S1 Table List of antibodies for flow cytometry. (PDF)

S2 Table Mutation status of PREACH-M cohort (*n* = 24) with regards to *CBL* and other RAS pathway mutations (*KRAS*, *NRAS*, *PTPN11*). (PDF)

S3 Table Spleen craniocaudal length (cm) of *CBL* mutant CMML vs RAS pathway wildtype. (PDF)

S4 Table Measurement of C_α_-C_α_ distance between residue 227 and 371^δ^ from structural analysis of Cbl wildtype and mutant proteins. (PDF)

S1 Fig Clinical characteristics of PREACH-M cohort at baseline, stratified according to *CBL* mutants and other RAS pathway (*KRAS*, *NRAS*, *PTPN11*) mutants *vs*. wildtype. (TIF)

S2 Fig VAF data of *CBL* mutant cases compared to *TET2* and other RAS pathway genes. (TIF)

S3 Fig Cytokine receptor CD114, CD115, CD116 and CD131 expression of CBL mutant and wildtype compared to healthy control by (A) percentage positive cells and (B) mean fluorescence intensity (MFI).
(TIF)

S1 Data (XLSX)
