# Supplementary material for: CBL mutations in chronic myelomonocytic leukemia often occur in the RING domain with multiple subclones per patient: Implications for targeting
Source: PLoS One. 2024 Sep 19;19(9):e0310641. doi: 10.1371/journal.pone.0310641 (PMC11412512; doi:10.1371/journal.pone.0310641)
Supplement: S1 Table — (PDF) [file pone.0310641.s001.pdf]

S1 Table. List of Antibodies for Flow Cytometry

Antibody	Clone	Manufacturer	Cat #
BV421 Mouse anti-human CD45	HI30	BD Horizon	563879
PE-Cy™7 Mouse anti-Human CD14	M5E2	BD Pharmingen™	557742
PE-Cy™5 Mouse anti-Human CD16	3G8	BD Pharmingen™	561725
APC Mouse Anti-Human CD34	8G12	BD	340441
PE Mouse anti-Human CD114	LMM741	BD Pharmingen™	554538
PE Rat anti-Human Hu CD115	9-4D2-1E4	BD Pharmingen™	565368
PE Mouse anti-Human CD116	hGMCSFR-M1	BD Pharmingen™	551373
PE Mouse anti-Human CD117	YB5.B8	BD Pharmingen™	555714
PE Mouse anti-Human CD131	1C1	Invitrogen	12-1319-42
BV21 Mouse anti-Human CD131	3D7	BD OptiBuild^{TM}	752992
