# Supplementary material for: CBL mutations in chronic myelomonocytic leukemia often occur in the RING domain with multiple subclones per patient: Implications for targeting
Source: PLoS One. 2024 Sep 19;19(9):e0310641. doi: 10.1371/journal.pone.0310641 (PMC11412512; doi:10.1371/journal.pone.0310641)
Supplement: S2 Table — (PDF) [file pone.0310641.s002.pdf]

S2 Table. Mutation status of PREACH-M cohort (*n*=24) with regards to *CBL* and other RAS pathway mutations (*KRAS*, *NRAS*, *PTPN11*).

	RAS pathway wildtype	RAS pathway mutant	
***CBL* wildtype**	6	7	13
***CBL* mutant**	6	5	11
