# Supplementary material for: CBL mutations in chronic myelomonocytic leukemia often occur in the RING domain with multiple subclones per patient: Implications for targeting
Source: PLoS One. 2024 Sep 19;19(9):e0310641. doi: 10.1371/journal.pone.0310641 (PMC11412512; doi:10.1371/journal.pone.0310641)
Supplement: S3 Table — (PDF) [file pone.0310641.s003.pdf]

S3 Table. Spleen craniocaudal length (cm) of *CBL* mutant CMML vs RAS pathway (*KRAS*, *NRAS*, *PTPN11*) wildtype.

		Spleen length (cm)	Splenomegaly[#]
CBL mutant	MEL13	12.6	Yes
	ADE09	17.4	Yes
	ADE17	16.3	Yes
	BRI07	20.6	Yes
	MEL05	12.5	Yes
	ADE02	13.2	Yes
	ADE03	13.0	Yes
	ADE20	18.8	Yes
	ADE21	20.3	Yes
	MEL06	10.2	No
	ADE23	12.0	Yes
		Total with splenomegaly	***10/11 (91%)***
RAS pathway wildtype	BRI14	10.8	No
	MEL18	11.9	No
	ADE10	13.8	Yes
	ADE22	10.9	No
	BRI12	12.9	Yes
	MEL24	11.5	No
		Total with splenomegaly	***2/6 (33%)***

[#]Splenomegaly defined as spleen cradiocaudal length equal or greater than 12.0 cm

Fisher's exact test; P<0.05 was statistically significant. P=0.0276.
