# Supplementary material for: CBL mutations in chronic myelomonocytic leukemia often occur in the RING domain with multiple subclones per patient: Implications for targeting
Source: PLoS One. 2024 Sep 19;19(9):e0310641. doi: 10.1371/journal.pone.0310641 (PMC11412512; doi:10.1371/journal.pone.0310641)
Supplement: S4 Table — (PDF) [file pone.0310641.s004.pdf]

S4 Table. Measurement of C_α-C_α distance between residue 227 and 371[δ] from structural analysis of Cbl wildtype and mutant proteins

PDB ID	Cbl structure	C_α-C_α distance (Å)
2Y1M	WT closed, inactive	9.8
4A4C	WT open, active	46.4
5HKX	Y371E mutant	16.9
5J3X	Y371F mutant	9.5

[δ]Residues 227 (in the TKBD) and 371 (in the LHR) forms a hydrogen bond in Cbl wildtype closed inactive state, used here as points of reference to show the displacement of LHR following phosphorylation or mutation [C_α alpha-Carbon, WT wildtype, TKBD tyrosine kinase binding domain, LHR linker helix region]
