# Supplementary figures and images for: CBL mutations in chronic myelomonocytic leukemia often occur in the RING domain with multiple subclones per patient: Implications for targeting
Source: PLoS One. 2024 Sep 19;19(9):e0310641. doi: 10.1371/journal.pone.0310641 (PMC11412512; doi:10.1371/journal.pone.0310641)

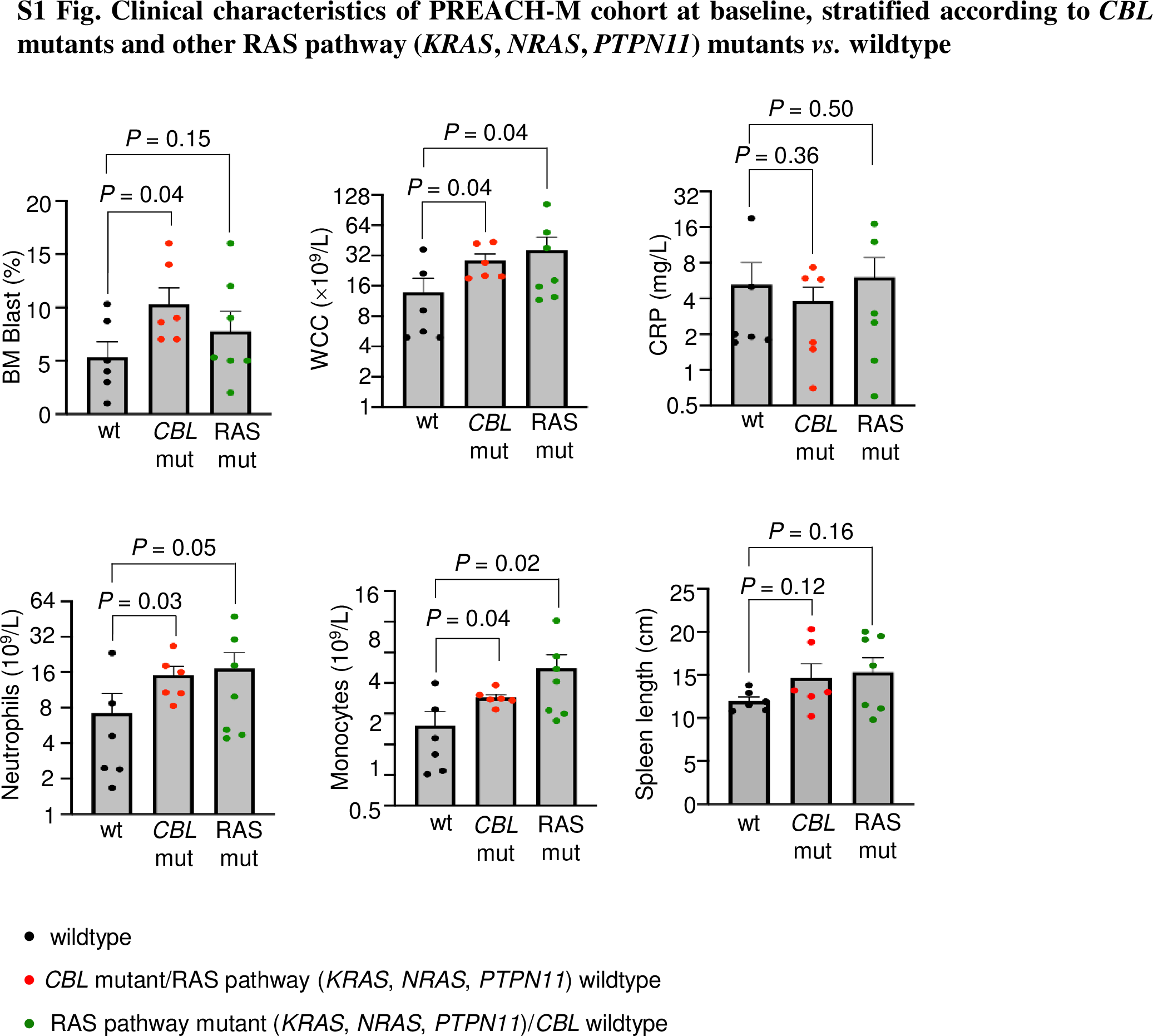

Supplement: S1 Fig — (TIF) [file pone.0310641.s005.tif]

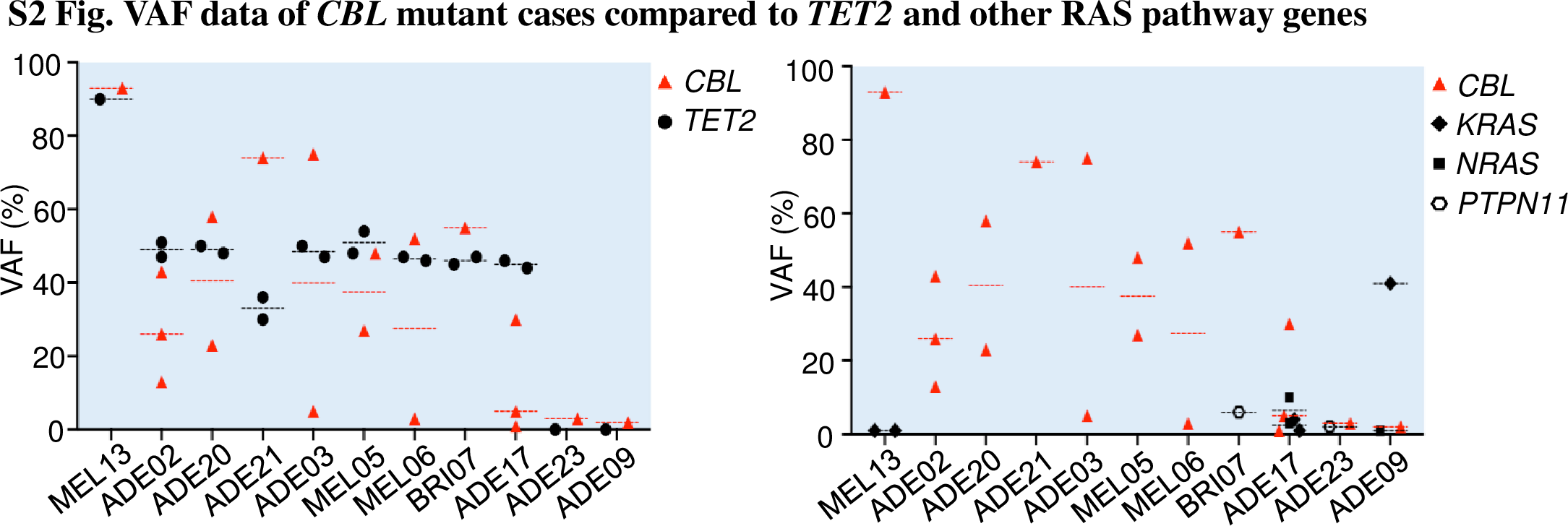

Supplement: S2 Fig — (TIF) [file pone.0310641.s006.tif]

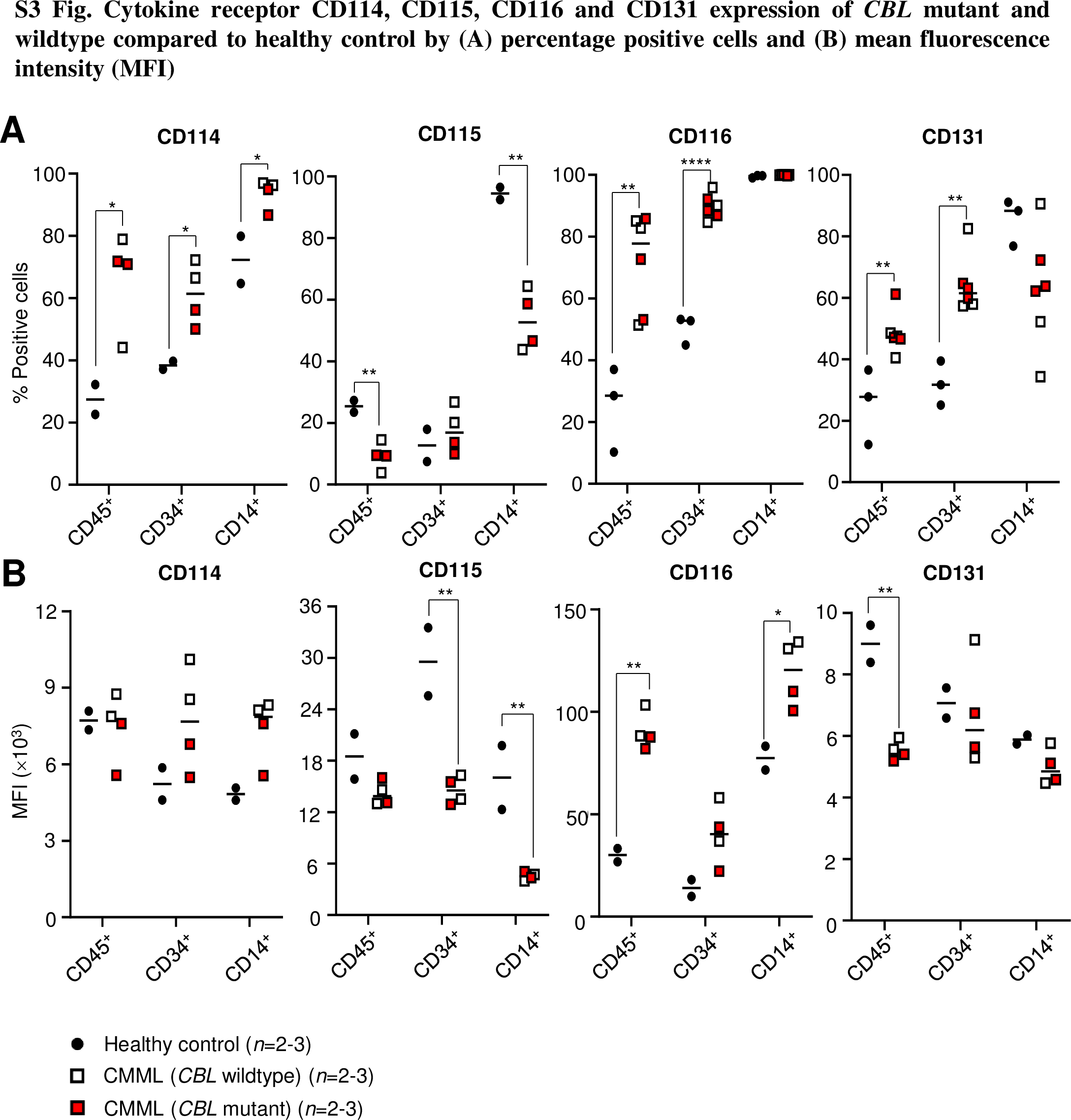

Supplement: S3 Fig — Cytokine receptor CD114, CD115, CD116 and CD131 expression of CBL mutant and wildtype compared to healthy control by (A) percentage positive cells and (B) mean fluorescence intensity (MFI). (TIF) [file pone.0310641.s007.tif]
